# The cult of amphioxus in German Darwinism; or, Our gelatinous ancestors in Naples’ blue and balmy bay

**DOI:** 10.1007/s40656-014-0034-x

**Published:** 2015-01-08

**Authors:** Nick Hopwood

**Affiliations:** Department of History and Philosophy of Science, University of Cambridge, Free School Lane, Cambridge, CB2 3RH UK

**Keywords:** Amphioxus, Edward John Chapman, Ernst Haeckel's Darwinism, Naples, Moritz Reymond, Humorous songs

## Abstract

Biologists having rediscovered amphioxus, also known as the lancelet or *Branchiostoma*, it is time to reassess its place in early Darwinist debates over vertebrate origins. While the advent of the ascidian–amphioxus theory and challenges from various competitors have been documented, this article offers a richer account of the public appeal of amphioxus as a primitive ancestor. The focus is on how the ‘German Darwin’ Ernst Haeckel persuaded general magazine and newspaper readers to revere this “flesh of our flesh and blood of our blood”, and especially on *Das neue Laienbrevier des Haeckelismus* (The new lay breviary of Haeckelism) by Moritz Reymond with cartoons by Fritz Steub. From the late 1870s these successful little books of verse introduced the Neapolitan discoveries that made the animal’s name and satirized Haeckel’s rise as high priest of its cult. One song is reproduced and translated here, with a contemporary “imitation” by the Canadian palaeontologist Edward John Chapman, and extracts from others. Predating the American “It’s a long way from amphioxus” by decades, these rhymes dramatize neglected ‘species politics’ of Darwinism and highlight the roles of humour in negotiating evolution.

From links in the great chain to model organisms, from the exotic to the domesticated, researchers have long placed special species on royal roads to knowledge. Evolutionists prize living fossils that might evidence the major transitions, such as the lancet-shaped sand-dweller of a few centimetres that in 1834 the Naples naturalist Oronzo Gabriele Costa recognized as a simple fish (De Ceglie 1999, pp. 87–99; Fortey 2011). From the 1860s the ‘German Darwin’ Ernst Haeckel celebrated amphioxus, also known as the lancelet or *Branchiostoma lanceolatum*, as the primitive ancestor of all vertebrates because, though limbless and headless, it contains an axial rod or notochord. The Russian embryologist Alexander Kowalevsky’s discovery, again in Naples, that it develops like the swimming larvae of the ascidians, or sea squirts, let Haeckel bridge the invertebrate–vertebrate divide (Beeson 1978; Mikhailov and Gilbert 2002; Fokin and Groeben 2008, pp. 99–102; Fokin 2012). Widely studied as the elementary vertebrate around 1900, amphioxus went out of fashion with the mid-twentieth-century decline of comparative anatomy and embryology. The renaissance of ‘evolution and development’ revived interest in what are now recognized as three genera containing some thirty species. Promoted by genome sequencing from nearly-vertebrate to basal chordate, the larger category of notochord-containing animals, the lancelet was “set to re-enter public life” (Gee 2008, p. 999; also Putnam et al. 2008; Bertrand and Escriva 2011). When the daily press reports as news that not just “[s]upermodels”, but all backboned forms “owe their body shapes to a limbless, headless fish-like creature likened to an ‘animated anchovy fillet’”,^1^ it is high time to re-examine those first years of fame.

Disputes over vertebrate origins are notorious for the variety of putative ancestors that challenged amphioxus and ascidian since Anton Dohrn, Haeckel’s estranged student and founder of the Naples Zoological Station, hypothesized changes in function that pointed to direct descent from annelid worms (Dohrn 1875; Kühn 1950; Ghiselin 1994; Maienschein 1994; Caianiello 2014; also Nyhart 1995, pp. 243–277). But survey histories have concentrated on specialists and, by chasing new ideas, have over-emphasized minority views (Gee 1996, pp. 84–159; Bowler 1996, pp. 141–202). We know that “[t]he theme of progress and degeneration … permeated the debate” (Bowler 1996, p. 146), but little about even the most prominent ancestor’s public life. To reveal how amphioxus caught the imagination when Haeckel persuaded general readers to revere that venerable beast as “flesh of our flesh and blood of our blood” (Haeckel 1874, p. 340), this article goes beyond his own works, monographs and journal articles to magazines, newspapers and, especially, books of songs and cartoons.

The soundtrack to the recent revival, “It’s a long way from amphioxus”, was written in the US in 1921 to the tune of the World War anthem “Tipperary”, and expresses by then conventional affection.^2^ Four decades earlier, and coming out of a still older German tradition of science and song (Jackson 2003), rhymes by Moritz Reymond with sketches by the caricaturist Fritz Steub calmed a bitter confrontation over “Haeckelism”. They celebrated the discoveries of what a free English translation called “our gelatinous ancestors” “in Naples’ blue and balmy bay” (Agorastes 1878), and satirized Haeckel’s rise as high priest of an amphioxus cult. They dramatize neglected ‘species politics’ of Darwinism and highlight the roles of humour in negotiating evolution.^3^

Illustrated songs about German scientists studying vertebrate origins in Naples are a fitting topic with which to honour Christiane Groeben, who has not only contributed much to our understanding of the Zoological Station and debates involving Dohrn. She has also kept alive his tradition of cultivating the arts in relation to the fauna and flora of the gulf (e.g., Groeben and Gambi 1992; Groeben 1995), including in memorable musical performances at the Ischia Summer Schools.

## Flesh of our flesh

In 1834 Costa, a professor of zoology embarking on a big study of the rich fauna of the Kingdom of Naples, reclassified what, from a preserved Cornish specimen, the German zoologist Peter Simon Pallas had 60 years before described and depicted as a mollusc. Costa placed live animals from Posillipo, just north of the city, as relatives of cyclostomes, the jawless hagfish and lampreys, though dissection soon showed that amphioxus deviated more from these than they did from salamanders (Willey 1894, pp. 7–9). Costa’s interest in links between classes and Europe-wide discussion as an intermediate form (De Ceglie 1999, pp. 87–99) confirmed amphioxus as the most primitive vertebrate (Gegenbaur 1859, pp. 386–393). In the 1860s the Jena zoologist Ernst Haeckel, with his anatomist colleague Carl Gegenbaur, evolutionized the science of form (Nyhart 1995; Richards 2008; Hopwood 2015, pp. 61–88) and gave it an even bigger part.

Haeckel filled the first Darwinist system, his *Generelle Morphologie* (General morphology), with cosmic ambition, new technical terms, ferocious polemic, bold pedigrees all the way up to human beings and taxonomic revisions which promoted amphioxus to a new subphylum, the Acrania or skull-less ones. As the single survivor of the first vertebrate division, Haeckel pronounced it “probably the last Mohican” of a once “richly developed and many-branched tree” (Haeckel 1866, II, p. cxix). The reference to James Fenimore Cooper’s novel invoked imperialist elegies for the last representatives of lineages, tribes and species (Stafford 1994, pp. 232–260; Brantlinger 2003), but Haeckel in fact picked winners, stressing the nobility of amphioxus as an ancestor of the dominant line and the respect owed to age. The transition from the invertebrates to amphioxus remained mysterious, however; he adopted a marine roundworm as a hypothetical link, but there was as yet no convincing bridge.

**Fig. 1:**
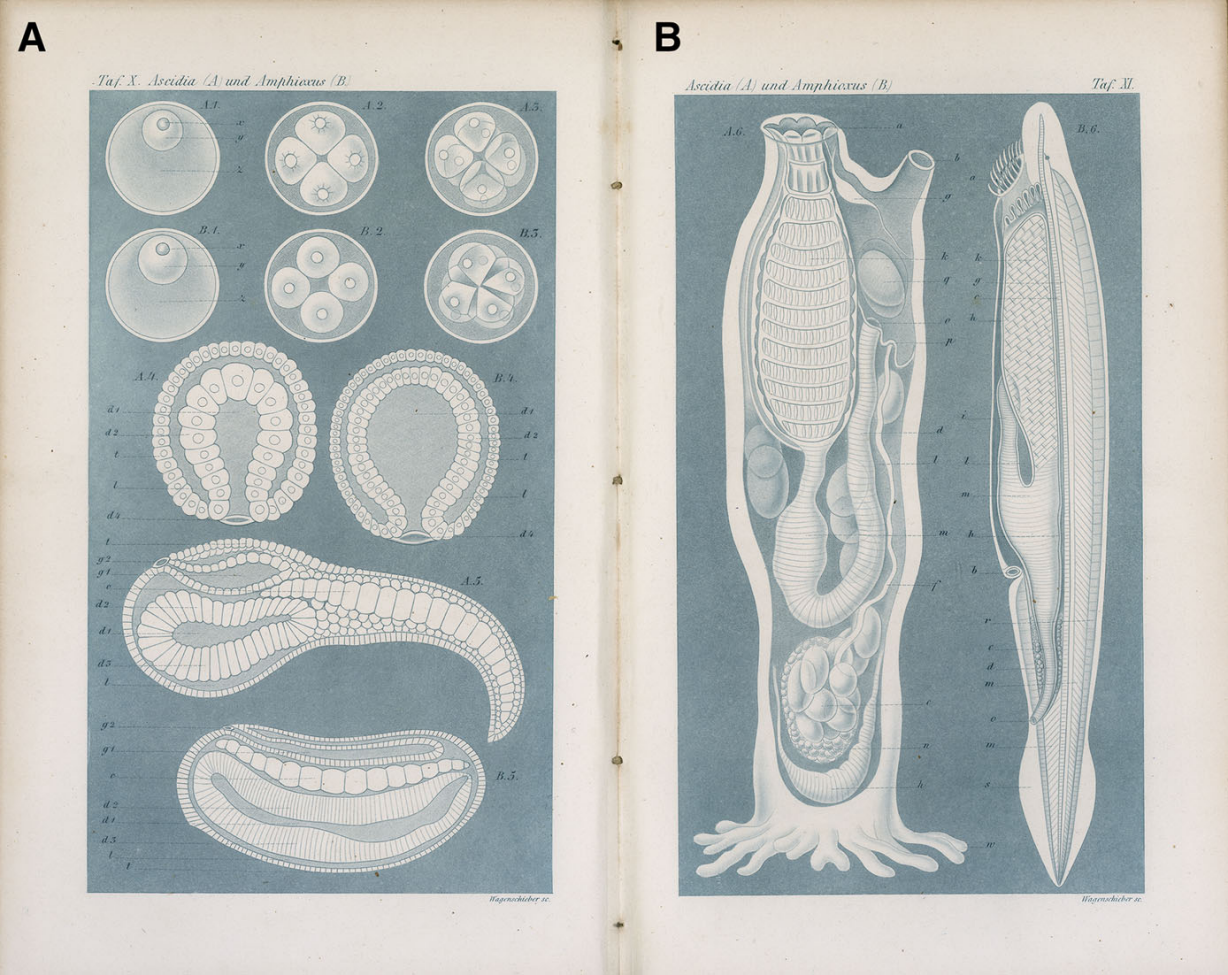
Plates of the embryology and anatomy of ascidian (**A**) and amphioxus (**B**) in the second edition of Haeckel’s *Natürliche Schöpfungsgeschichte* to show the “blood relationship of vertebrates and invertebrates”. Simplified after Kowalevsky, *plate X* represents the ontogeny in five stages from eggs to larvae, and *plate XI* the adults with the internal organization visible through the transparent skin. Copper engravings with blue wash by Wilhelm Wagenschieber from Haeckel (1870, plates X–XI). 21 × 26 cm

From 1868 Haeckel’s *Natürliche Schöpfungsgeschichte* (Natural history of creation) preached this Darwinist gospel and, over several decades, a dozen German editions and as many translations took it to the general educated public. Lacking fossils, his conjectural pedigrees relied on embryos, via the theory that individual development (ontogeny) recapitulates that of the species (phylogeny). Still cautious about vertebrate origins in 1866, Haeckel now used Kowalevsky’s work to argue that an invertebrate like an ascidian larva, itself derived from annelids, had developed in two directions: degenerating into the adult tunicates sitting on the sea floor and progressing to amphioxus and other vertebrates (Haeckel 1868a, pp. 409, 433–440). This story raised the credibility of the lancelet too. “We must thus contemplate amphioxus with special reverence”, a “popular scientific lecture” proclaimed, “as that venerable animal which of all still living animals is alone in a position to give us an approximate idea of our oldest Silurian ancestors with backbones” (Haeckel 1868b, p. 42). Fine schematic plates of ascidians and amphioxus graced the second edition of the *Schöpfungsgeschichte*, bridging a “deep gulf” and allowing Haeckel to trace humans back to worms (Fig. 1). He told “speculative philosophers” to stop “building futile castles in the air” and think about these facts instead (Haeckel 1870, p. 673). Darwin’s *Descent of Man* less polemically promoted the ascidian–amphioxus theory the following year (Darwin 1871, I, pp. 204–206). Humble origins were an old idea—liberals cared only how far a person had come and could go—but the specificity and degree of consensus were new.

Haeckel’s pedigrees stirred up trouble from the start, but editors, journalists and general readers entrenched the *Schöpfungsgeschichte* in the optimistic, nationalist, anticlerical ‘culture of progress’ of the new German Empire and took these ancestors into middle-class homes.^4^ Then, in 1874, Haeckel came out with the *Anthropogenie* (translated as *The Evolution of Man*), a major statement of the relations between ontogeny and phylogeny and a salvo in the *Kulturkampf*, Chancellor Bismarck’s anti-Catholic campaign (Haeckel 1874, pp. xi–xvi). Joining the nationalist monument-building (Belgum 1998, pp. 84–102; Jefferies 2003, pp. 59–72), the book proudly traced humanity back to amoeba, amphioxus, axolotl, Australian lungfish and ape (Hopwood 2015, pp. 108–119). Haeckel told readers that “ignorant theologians” and “anthropocentric” philosophers objected that his attitude towards amphioxus had “ridden roughshod over … the dignity of humanity and most grievously insulted the divine human sense of reason”. But, he insisted, the “thousand-year-old oak wood” it seemed natural to respect was as nothing compared to this little fish. If amphioxus lacked “skull, brain and limbs”, this “flesh of our flesh and blood of our blood” still deserved more admiration and reverence “than all the useless rabble of so-called *saints* to whom our ‘highly civilized’ nations build temples and dedicate processions” (Haeckel 1874, pp. 337, 340). Haeckel deployed fresh empirical research and his own new argument that all true animals descended from a swimming stomach, or *gastraea*, to strengthen and expand his coverage of the ascidian–amphioxus theory (Beeson 1978, pp. 335–356). He mobilized these animals as secular icons and in a struggle for embryology between phylogeny and physiology that politicized species choice (Hopwood 2011, p. 5). One look at amphioxus development, he declared, and the chick-based theory of his rival, Wilhelm His, “collapses like a house of cards” (Haeckel 1874, p. 629; also 1875, p. 23).

**Fig. 2:**
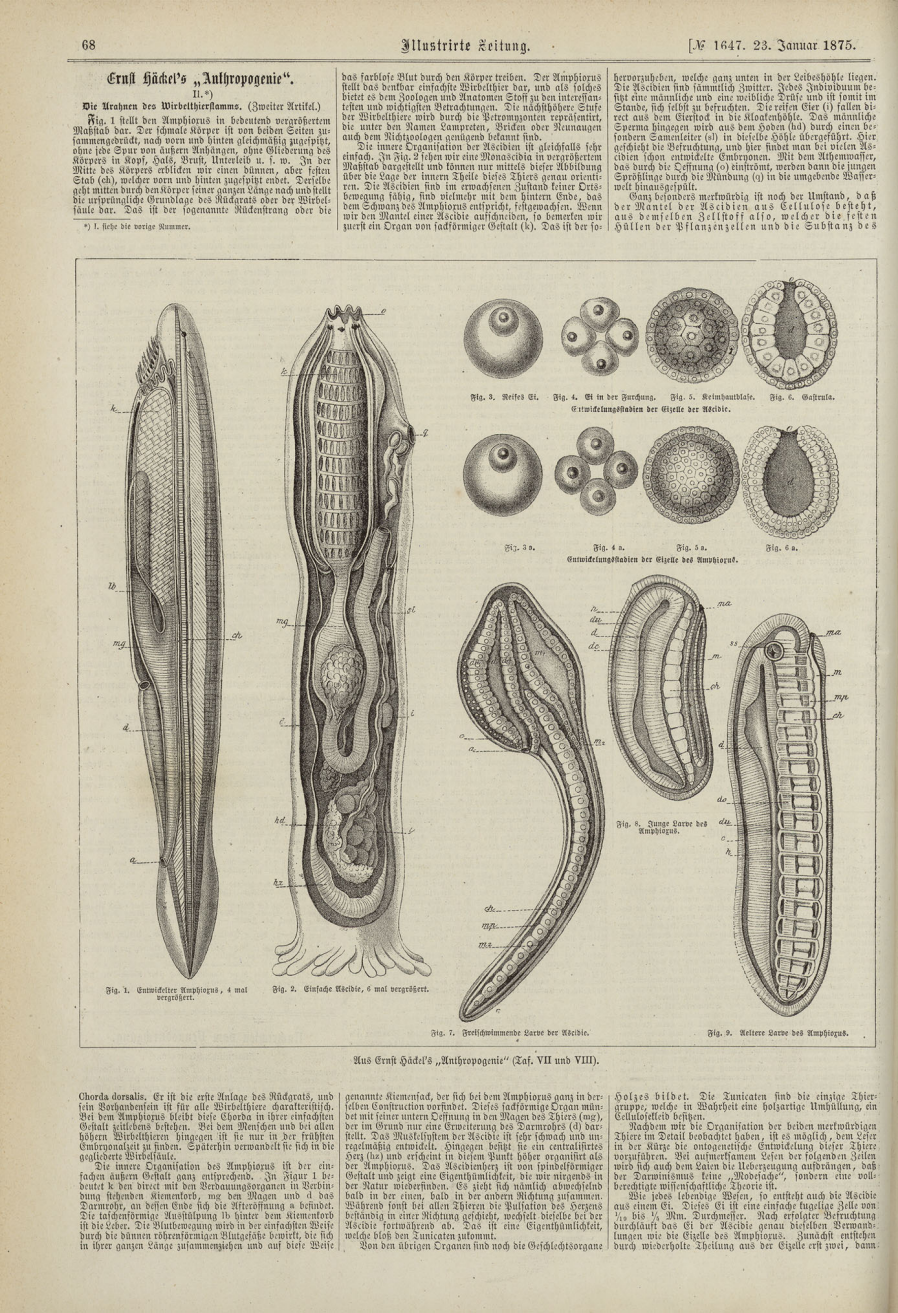
Amphioxus and ascidian in the *Illustrirte Zeitung*. These wood engravings in Otto Zacharias’s review of Haeckel’s *Anthropogenie* are cruder than the original plates but reached more people. The magazine omitted cross-sections and a lamprey larva that Haeckel had added to present his more developed theory in the more specialized book. Zacharias (1875a, p. 68). 41 × 29 cm. Niedersächsische Staats- und Universitätsbibliothek Göttingen

More specialized than the *Schöpfungsgeschichte*, and hard for those Haeckel disparaged as “so-called educated” graduates of the classical grammar schools (Haeckel 1874, p. xi), the *Anthropogenie* disappointed high expectations of a good read, but many learned the arguments at second hand (Hopwood 2015, pp. 127–134). The liberal publicity machine announced the book with a review in the *Illustrirte Zeitung* (Illustrated news) that told progressives to study comparative embryology. Large wood engravings presented amphioxus and ascidians as “primordial ancestors of the tribe of backboned animals” (Fig. 2).^5^ *Die Gartenlaube* (The bower), the family magazine with the huge run of nearly 400,000, also stressed their significance as a bridge. Without the “little lancet fish” we would barely know we came from worms, a “kinship certainly more congenial to the Christian feeling of humility than descent from the apes”.^6^ But the *Anthropogenie* did not just give journalists material; it also goaded experts into warning the public (Hopwood 2015, pp. 119–126, 132–134).

Haeckel basked in adulation, but his polarizing rhetoric also provoked howls of opposition from scientists as well as philosophers and theologians. A disciplinary enemy, the physiologist Emil du Bois-Reymond, had already attacked his overstepping of the bounds of natural knowledge (Bayertz et al. 2007). Even fellow zoologists, most of whom by now accepted the fact of evolution, worried about the dogmatic presentation of personal speculations, exaggeration of his own novelty and intolerance towards other researchers’ views. Few Darwinists endorsed every detail of his pedigrees and many objected to his gastraea theory (Nyhart 1995, pp. 168–206). Yet amphioxus had until now largely escaped specialist challenge.

Haeckel’s advocacy and Darwin’s endorsement had established the lancelet as the primordial vertebrate and built momentum behind the more controversial ascidian theory, but from 1875 other Darwinist theories began to compete (Beeson 1978).^7^ From Naples, Anton Dohrn urged followers of Haeckel and Gegenbaur to pay living conditions more attention. Then they would see that ascidians and amphioxus merely continued the degeneration that the parasitic cyclostomes had begun. Reduced to filtering food from sandy water, amphioxus represented “a degenerate fish”, and the ascidians had declined even more (Dohrn 1875, p. 55; Ghiselin 1994, p. 63). For the Würzburg zoologist and authority on sea cucumbers Carl Semper, the other main advocate of the annelid theory, amphioxus was also no vertebrate (Semper 1875, p. 66; 1876a). This comment in Semper’s institute journal gained currency when the veteran materialist and early Darwinist Carl Vogt deployed it in the liberal *Frankfurter Zeitung* to dismiss Haeckel’s “mythical” pedigrees, and anti-Darwinists picked it up.^8^ Few were in any position to adjudicate, but expert disagreement made the choices look arbitrary and helped those who rejected pedigrees out of hand.

Haeckel defended himself, in a small section of a long polemic, by pretending that for consistency Dohrn would have to make the whole animal kingdom degenerate from the “sinless” Adam until the “unfortunate amphioxus, which had burdened itself with the most serious guilt, finally lost even its head” (Haeckel 1875, pp. 31–33, 88–89). But as religious and political protests rained down on Haeckel, and the press amplified colleagues’ censure of everything from pictures of embryos to speculations about cellular souls, objections to amphioxus as ancestor were still easily overlooked. For spokesmen of science the bigger issue was the threat from his behaviour to their freedom in the new state. In early 1876 Semper publicly targeted Haeckelism for falling into the same credulous dogmatism that its author repudiated in the churches, and so risking the status that Darwinism had brought zoology, but he mentioned amphioxus only in a note (Semper 1876b, p. 35).

Haeckel’s reputation would never recover fully from these battles, but most competent researchers still credited him for pushing the theory of evolution through. As tempers frayed, humour, which had so far figured mostly in sarcasm between Haeckel and his critics, appeared as mediator. Rhymes went some way towards defusing a tense situation by separating Haeckel’s excesses from commitments on which evolutionists just might agree.

## Haeckelism in rhymes

Nature researchers in the German lands had long sung together to foster (male) camaraderie and cultural nationalism, validate specialized knowledge (Jackson 2003), and share the fun in songs such as Joseph Victor Scheffel’s mock-tragic lament by the last ichthyosaur. A commission from the Bern scientific society spurred the newspaper editor Moritz Reymond to write humorous verses on intellectual fashions such as the *Kulturkampf* and medical advice, and Haeckelism became his greatest theme.^9^ With a title referring to Catholic books of daily prayers and the like for non-priests, and a focus on the Old Testament, *Das neue Laienbrevier des Haeckelismus* (The new lay breviary of Haeckelism) eventually ran to five books with cartoons by Fritz Steub of the *Fliegende Blätter*, the Bavarian *Punch*.

Reymond prepared *Genesis*, *oder die Entwickelung des Menschengeschlechts* (Genesis, or the development of the human race) for Christmas 1876. Shrinking the octavo lectures of the *Anthropogenie* chapter by chapter to a sexto-decimo of songs painlessly acquainted readers with the positive and negative sides of the original. Engaging with its reception too, the satire played on the symmetry between Haeckel’s charges of ignorance and the exclusivity of his own neologisms, on the one hand, and the irony of his condemning church dogma while setting himself up as the “apostle of a new faith”, on the other (Reymond 1877, p. 8). Reymond tackled the pedigrees at length, but left the hypothesis of amphioxus as ancestor unchallenged; the consensus was too strong and dissent not yet prominent enough. Two songs reviewed the discoveries that had revealed respectively the structure of amphioxus and ascidians and their embryology and thematized the German tradition of finding origins in and around the Mediterranean.

**Fig. 3:**
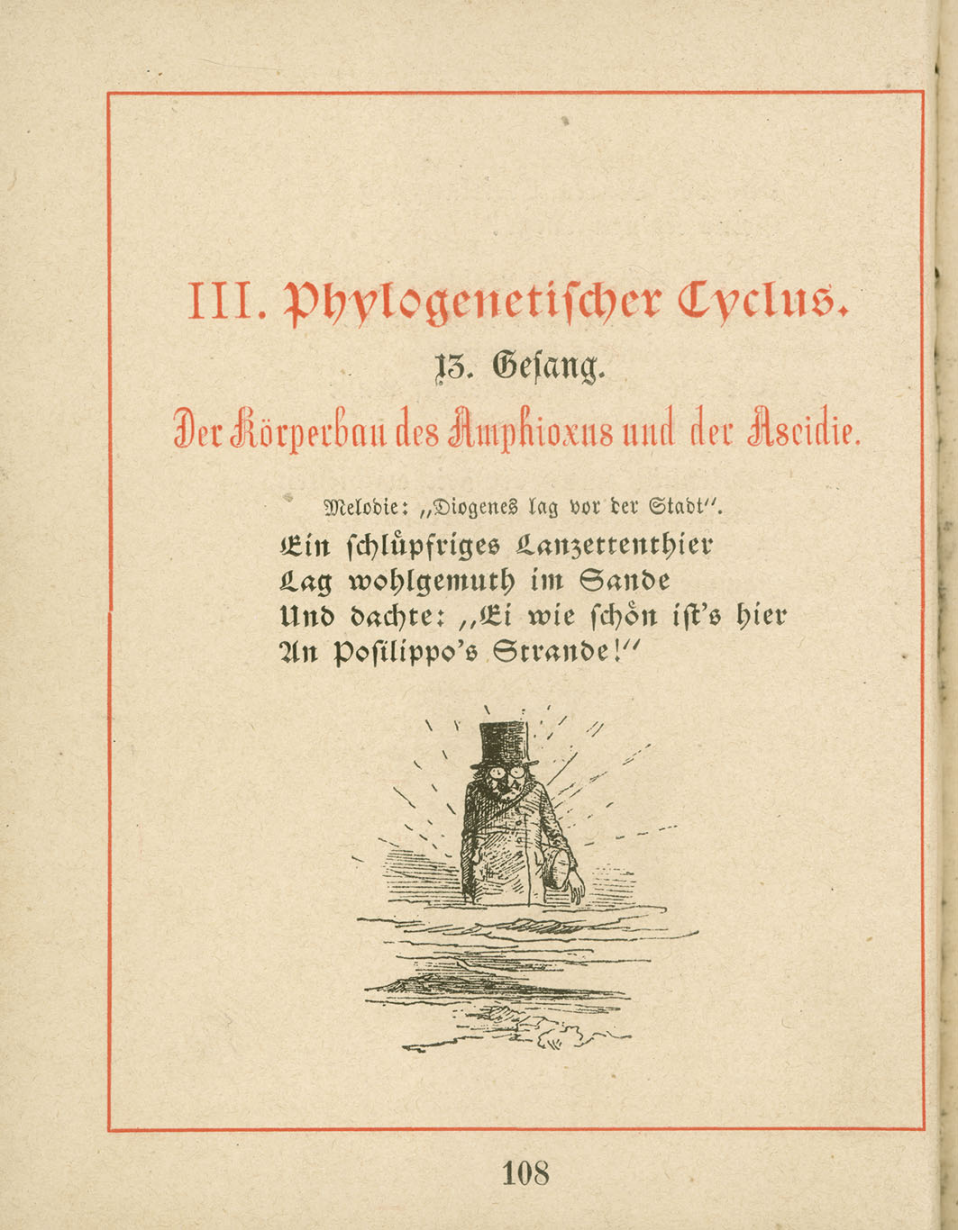
A “grim zoologist” (Costa) “on the horizon”. The illustration by Fritz Steub, like the one in Fig. 4, was added in the second and kept in this third edition. Reymond (1878, p. 108). Border 10.5 × 8.2 cm

The first, to a folk tune previously used for a student song about Diogenes the Cynic and Alexander the Great, corresponded to Chapter 13 of the *Anthropogenie* and tells how zoologists’ lancets raised the status of the lancelet (Reymond 1877, pp. 76–79). A series of encounters with Costa, Kowalevsky and Haeckel, the extent of whose hands-on research is exaggerated, prove fatal for the individual animals. Invoking Pallas fails to stop Costa testing his hunch (Fig. 3). But the species gain fame. Here is the complete song, “Der Körperbau des Amphioxus und der Ascidie” (The structure of amphioxus and ascidian), with my translation but without the four endnotes that explained the history in quotations from the *Anthropogenie*.

**Table:** 

Ein schlüpfriges Lanzettenthier	A lancet fish so slippery
Lag wohlgemuth im Sande	Lay cheerfully in the sand
Und dachte: “Ei wie schön ist’s hier	And thought, “Oh, isn’t it lovely
An Posilippo’s^10^ Strande!”	Here on Posillipo’s strand!”
Da stieg ein grimmer Zoolog	But then a zoologist grim
Empor am Horizonte,	Rose up on the horizon,
Der Alles zu zergliedern pflog,	Who used to dissect with much vim,
Was er erwischen konnte.	Whatever he laid hands on.
Er sieht das Thier und hebt es auf,	He sees and snatches the creature
Obwol es schneckenglatt ist,	Though smooth as a snail it be,
Und sagt: “Ich nehme Gift darauf,	And says: “My own life I’d wager,
Wenn das kein Vertebrat ist!”	But vertebrate that must be!”
Das Thierchen seufzt: “Ach, geh’n Sie weg!	The animal sighs: “Be off, you!
Das könnt’ ein Jeder sagen!	Anybody could say that!
Ich bin ja nur ein nackter Schneck—	A slug is all I am, it’s true—
Sie können Pallas fragen!”	Just go and ask Pallas that!”
Der Zoologe aber sprach	The zoologist however spake
Zum großen Schreck des Findlings:	To great terror in his find:
“Der weise Mann sieht selber nach	“The wise man must his own look take
Und glaubt nicht Andern blindlings!”	And doesn’t trust others blind!”
Drauf schob die Pseudoschnecke er	With that the pseudo-snail he tucked
Gefühllos in die Tasche,	Callously in his pocket,
Zerschnitt und setzt’ sie hinterher	Cut it to bits and these then dunked
In eine Weingeistflasche.	In a bottle of spirit.
Dies kam, wie sehr auch paradox	However great a paradox
Es scheint, dem Thier zu Statten;	This seems, the beast uprated;
Denn seither zählt der Amphiox	For since that day ranks amphiox
Zur Crême der Vertebraten.———	As crême of the vertebrated.———
Und wieder schwamm im Mittelmeer	Again a zoological
Ein Zoolog spazieren,	Went for a swim in the Med,
Wo die Ascidien stationär	Where ascidians statical
Am Grunde vegetiren.	Vegetate on the seabed.
Mit einem Schleppnetz und Bedacht	With drag-net, care and attention
Hat er an jenem Orte	He in that same place did bring
So manches Stück zu Tag gebracht	To light a splendid selection
Von dieser Würmersorte.	Of this type of wormy thing.
Es barg der Cellulose-Schlauch	The cellulose tunic it held
Die wunderlichen Alten	The wondrous adults mature
Und gleich die lieben Kleinen auch	And the dear babies also dwelled
In der Cloake Falten.	In cloacal folds secure.
Sie kamen unter’s Mikroskop,	They came under the microscope,
Sie kamen unter’s Messer;	They came under the cutter;
Sie starben sich zu Tod’ darob—	They died a death without a hope—
Doch Häckeln ging es besser!	But Haeckel’s life went better!
Denn sieh! Ein Wunder hatte da	For see! A miracle had there
Enthüllt mit einem Mal sich:	Revealed itself once for all:
Die Larve der Ascidia	The larva of ascidia
Erwies als vertebral sich!	Turned out to be vertebral!
Der Häckel hört’s von ungefähr	Haeckel heard, by chance ’twas really,
Und schnürte gleich den Ranzen	And packed his bags right away
Und ging an’s Mittelländ’sche Meer	And went down to the Middle Sea
Drei Monat in Vakanzen.	For three months of holiday.
Es forscht’ der große Zoolog	That zoologist searched the ground
Von früh bis Abends spät hier	From early till evening late
Und fand, wie sehr doch analog	And most analogous he found
Ascidie und Lanzett-Thier.	Ascidian and lance-let.
Was ihm gemangelt bis anhin	What till then he had been lacking
In seiner Ahnenreihe,	In his ancestral series,
Das Bindeglied nach unten hin—	The downwards link a-connecting—
Das waren diese zweie!	That was this pair of beasties!
Nun war die Lücke ausgefüllt,	Now the gap it was nicely healed,
Die Reihe schön geschlossen,	The series well closed henceforth,
Und das Geheimniß uns enthüllt,	And the secret to us revealed,
Wie wir dem Wurm entsprossen!	How from the worm we sprang forth!
Ascidie und Amphiox,	Ascidian and amphiox,
Ein Glas zu Eurem Ruhme!	Let’s raise a glass to your fame!
Den Wurzeln unsers Ahnenstocks	The stem of our ancestral stocks
Bring Jeder seine “Blume”!	Let everyone toast your name!

Listeners were invited to drink to their newest-oldest ancestors.

**Fig. 4:**
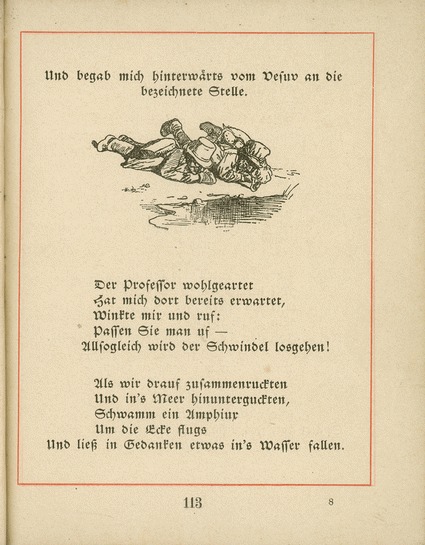
Alexander Kowalevsky and Brother Straubinger watching amphioxus mating in Naples. By Fritz Steub, from Reymond (1878, p. 113)

The next chapter, and so the next song, was about the embryology—presented through a chance meeting over a beer between Kowalevsky and “Brother Straubinger”, a travelling journeyman whose literary career had begun in the drinking song of a Bavarian medical student some 50 years before (Reymond 1877, pp. 79–83). There is space here only for a selection of verses in which Straubinger recounts what happened after the St. Petersburg professor invited him out. Kowalevsky, whose report described amphioxus as starting to mate on warm May evenings between seven and eight, asks his acquaintance to appear on the beach “behind Vesuvius”, “sober” and at half past seven precisely (Fig. 4).

**Table:** 

Der Professor wohlgeartet	The professor, good-humouredly,
Hat mich dort bereits erwartet,	Was there and waiting already,
Winkte mir und ruf:	Waved and gave a shout:
Passen Sie man uf—	Hallo, mate, look out—
Allsogleich wird der Schwindel losgehen!	The show is going to start any minute now!
Als wir drauf zusammenruckten	When we moved closer, he and me,
Und in’s Meer hinunterguckten,	And looked down, down into the sea,
Schwamm ein Amphiux	An amph’ux swimmer
Um die Ecke flug’s	Sped round the corner
Und ließ in Gedanken etwas in’s Wasser fallen.	And, in thought immersed, let something fall into the water.
Alldieweil traf unterwegs ihn	Where’pon an amph’oxy lady
Die Madame Amphiöxin;	Meeting him en route, silently
Dachte sich: “Oho,	Thought to ’self: “Wahey,
Steht die Sache so?”	Are things now that way?”
Und deponirte selbigenorts diverse Amphioxeneier.	And deposited various amphioxus eggs in the very same place.
Diese amphioxigen Eier	These eggs so amphioxical
Int’ressirten ungeheuer	Appeared of interest colossal
Kowalewsken, der	To Kowalevsky,
Alsbald hinterher	Who now directly
In’s Meer sprang und dieselben mit nach Hause nahm.	Jumped into the sea and took the same home with him.

Straubinger goes back to the Russian’s lodgings and they watch the amphioxus develop.

**Table:** 

Dorten konnten wir betrachten,	There we could contemplate steady
Wie bereits um Mitternachten	How around midnight already
Aus den Eiern zart	Those eggs so tender
Eine Blase ward,	Became a bladder
Welche Kowalewsky das Blastodermichen nannte.	Which Kowalevsky called the blastodermlet.

The uneducated artisan, who symbolized conflict with the academic world, has difficulty with “the damned Latin names”. Reymond thus both followed Haeckel and his readers in thematizing the difficulty of the esoteric science, and borrowed Straubinger’s idiom to communicate some embryology:

**Table:** 

Doch im weiteren Entwickeln	Yet as it develops quickly
Spürt die Larve nun ein Prickeln,	The larva now feels all tingly
Rücken auf und ab,	Up and down its bod’
Und ein Axenstab	And an axis rod
Wächst ihr bratspießartig mitten durch’s Gedärm.	Grows right through its intestines like a skewer.

Working up an appetite, Straubinger would not have minded hearing more over some spare ribs, “because you don’t never know what education will be good for”, but Kowalevsky sends him away hungry.

Admirers and critics of Haeckel liked the rhymes (Hopwood 2015, pp. 142–143). A Catholic reviewer was pleased to see Darwinism’s true colors exposed, and for an editor of the leading anti-Darwinist science magazine “this food for the mind” complemented the Christmas sweetness as perfectly as “herring salad with Spanish pepper … a stomach upset by marzipan”.^11^ Others saw Reymond as helping Haeckel, who was rumoured to have kept a copy in his jacket pocket (May 1909, p. 116), even though many verses cut nearer the bone than those about amphioxus. In Jena “some of the best bits … were read out before the … zoology practical”.^12^

English-speakers familiar with Haeckel’s books, and the debates over them, enjoyed Reymond’s verses too. In 1878 the British-born, Göttingen-educated Toronto palaeontologist and poet Edward John Chapman translated the first amphioxus song. His biologist colleague Robert Ramsay Wright had paid this animal much attention when reviewing the *Anthropogenie* for the local scientific society, the Canadian Institute. Like most professors, he found the book “admirably lucid” while regretting the “assertive tone”, “dogmatism” and “cavalier treatment” of opponents (Wright 1876). Under the pseudonym Dr. Agorastes (“marketeer”), perhaps taken from Lucian’s *Sale of philosophies*, Chapman dedicated his translation to Wright and the institute.^13^ But *Amphioxus and ascidian*, *our gelatinous ancestors* “imitated” as much as it translated; compare what follows with my more literal rendition above. Chapman added information, colour and drama, accommodated with an extra line in each of three fewer verses, as he turned a drinking song into a lyric poem, a pictureless “piece of jocose rhyming” in the style of Samuel Taylor Coleridge, to be declaimed more than sung.^14^

A slimy lancelet once lay

  Half-hidden in the golden sand

Of Naples’ blue and balmy bay—

And thought—how pleasant, here, to-day

  Is Posilippo’s strand.

But on the horizon’s shadowy brim

  A horrid vision doth arise—

A spectacled Professor grim!

All things that creep, crawl, fly, or swim,

  Must he anatomize!

A “find” his eager senses sniff—

  He stoops—he sees: with joy elate

He grasps the creature in a jiff—

And cries—now, I’ll be jiggered—if

  It isn’t vertebrate!

The victim groan’d—“Come, that’s *too* pale—

  Don’t try on me that precious cram—

Limpid and soft from top to tail,

I’m nothing but a naked snail—

  Ask Pallas what I am!”

The horrid “Zoo” made answer, “nay!

  I mean, my dear, myself to ‘fix’ it:

No wise man trusts what others say,

Or heeds, in this far-searching day,

  The dead Past’s *ipse dixit*!”

So saying, without more ado,

  (To tender feeling sadly callous)

He slit poor Slimy through and through,

And bottled—as he’d bottle you—

  This pseudo-snail of Pallas!

And thus, although the little nata-

  Torial beast has no backbone,

For reasons based on larval data

It came among the Vertebrata

  A place, at last, to own!

Pass thirty years and two—Ah me!

  How quick Time gallops!—Then there came

A learned Russ to that blue sea—

To fish for tunicates came he,

  Also to fish for fame!

And so before his zeal should flag,

  Or fall below its high meridian^15^—

To work he went with dredge and drag,

And fished up quite a thumping bag

  Of things ascidian!

And then came out the startling truth—

  Let all the world’s four corners hear it!

The ascidian in its frisky youth

Is half a vertebrate, in sooth,

  Or something somewhere near it!

Swift sped the news o’er land and sea,

  Till reaching Haeckel’s ears—that great

Stem-framer—in vacation free—

Pack’d up, and went post-haste to see

  This yea-nay vertebrate!

And there, our great Ontogenist—

  Whose word bewitches whilst it shocks us—

Beheld the links his System miss’d:

Yes, here they were—or he’d be hiss’d—

  Ascidian—Amphioxus!

All things are sure to one who waits:

  And here the links at last were seen

Made manifest to meanest pates—

Invertebrated Vertebrates—

  Fishes and worms between!

Thus, haeckelism’s wondrous gleam

  Makes clear, to all, how all arose—

Backward and forward went the stream

Of shifting forms, like shapes of dream,

  And found in us its close!

Chapman thus replaced the toast with a Romantic scene that perhaps foresaw endless revision to the pedigree.

## Hats off to amphioxus

Meanwhile, the German controversy over Haeckel raged on. In the *Neue Freie Presse* (New free press) of Vienna Vogt analysed Haeckel’s metamorphosis from “apostle” through “prophet” to “oracle”. Vogt ironically confessed as his own “gravest sin” that he had denied human kinship with ascidians and amphioxus and lampooned Haeckel’s “enthusiastic exaggeration”. He now protested against even more extreme “hyperbolicization” by exclaiming with reference to the demand of the Austrian tyrant defied by the Swiss patriot Wilhelm Tell: “How long will it be before we have an edict that we must doff our hats to a jar of amphioxus, like in front of Geßler’s pole with a hat on top!” (Vogt 1877). In Reymond’s verses, which developed this criticism, not long.

**Fig. 5:**
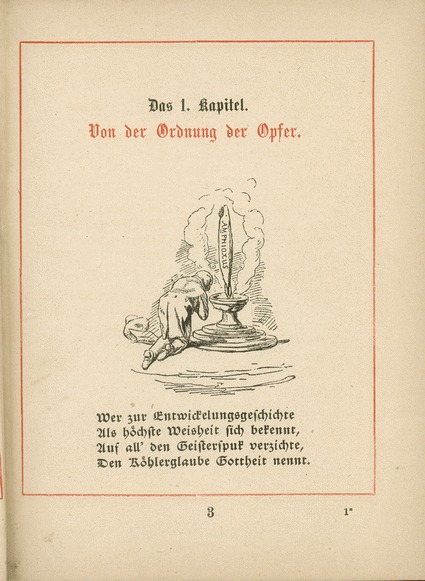
The cult of amphioxus in Moritz Reymond’s *Leviticus*: the high priest Haeckel kneels as he gives instructions on bringing offerings. By Fritz Steub, from Reymond (1880a, p. 3). Border 10.5 × 8.2 cm

Following the second book of the breviary, the more critical *Exodus*, a new instalment containing books three to five, *Leviticus*, *Numbers* and *Deuteronomy*, completed the Pentateuch in 1880. These still poked fun at the pedigrees, and now also satirized the latest debates by imagining the establishment of Haeckelism as the state religion and Haeckel’s installation as high priest of an amphioxus cult. *Leviticus*, or “the law for the prophets of the gospel of development”, opened with Steub’s sketch of a kneeling Haeckel (Fig. 5) and this verse (Reymond 1880a, pp. 3–4):

**Table:** 

Wer staunte nicht das edle Wesen	Who wouldn’t marvel with reverence,
Voll Ehrfurcht und Verwund’rung an,	Full of admiration most bold
Durch das die Wissenschaft genesen	For the noble one which freed science
Vom alten blinden Schöpfungswahn?	From blind creationism old?
Das unsers Menschenstammbaums Lücke	That golden bridge of discovery
Mit seiner Herrlichkeit erfüllt,	Which with glory magnificent
Als goldene Erkenntnisbrücke	Fills a great gap in man’s pedigree,
Uns den Entwicklungsplan enthüllt?!	And reveals our development?!

The singer argued that if psalms must be sung, then to this little fish; if offerings made, then to the first animal ever to develop vertebrae, a “headless-unconscious great deed of development” by the “ancestor and creator” of human beings. There should be no blood sacrifice, however. Haeckel was presented as tempted to do away with some senior professors—he disliked “Berlin biology”—but as choosing to give up dubious doctrines instead. In real life he maintained to the end, for example, the place of ascidians in the human stem-tree.

**Fig. 6:**
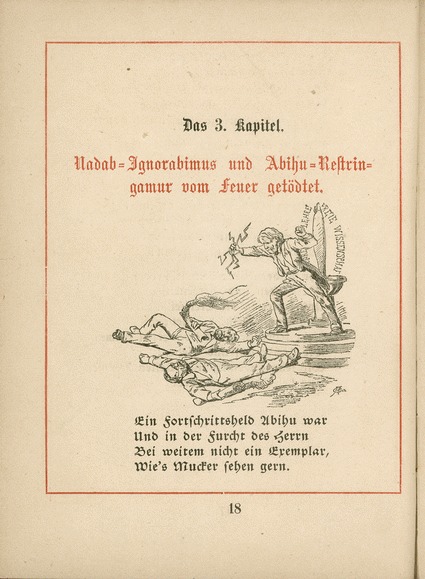
“Nadab-Ignorabimus and Abihu-Restringamur killed by fire”. Du Bois-Reymond had declared “Ignorabimus” (We shall not know): scientists would never solve certain riddles. Virchow had urged caution—“Restringamur” (Let us exercise restraint), as Haeckel put it—in order not to turn people against science. By Fritz Steub, from Reymond (1880a, p. 18)

If, when ordaining the Darwinist priests, Reymond’s Haeckel detected any concession to creationism and teleology or scepticism towards recapitulation, he felled them with a lightning bolt and finished them off with “bad jokes” (Reymond 1880a, pp. 10–17). His pamphlet on freedom in science and learning slaughtered the traitors Abihu, that is the left-liberal pathologist Rudolf Virchow, who had opposed the teaching of evolution in the schools, and Nadab, alias Reymond (1880a, pp. 18–25) (Fig. 6). Peace came when, like Yahweh quashing the rebellion of the tribes of Israel by causing Aaron’s rod to bud, Darwin ended his followers’ rivalry by keeping evergreen only Haeckel’s tree of “gastrae’, amphiox and ape”.^16^ Reymond parodied Haeckel’s cult, but reflected the consensus on the ancestral status of amphioxus.

## This exquisite form

Having subsided in the 1880s, Darwinist debate revived in the following decade. From 1899 Haeckel’s bestselling anticlerical synthesis *Die Welträthsel*, translated as *The Riddle of the Universe*, expanded the audience for science, took him to dizzying heights of international notoriety and fame, and created a market for a fresh English translation of *The Evolution of Man* (Hopwood 2015, p. 167). New readers also discovered the *Laienbrevier*, including in the US (Kellogg 1908). According to an “anniversary edition”, Haeckel recommended it to students too busy drinking and fencing for lectures (Reymond 1912, p. 251). He still praised amphioxus as “after man the most important and interesting of all vertebrate animals”, a claim his translator exaggerated by omitting the “vertebrate” (Haeckel 1903, II, p. 441; Haeckel 1905, II, p. 419; see also Haeckel 1874, p. 298; Haeckel 1899, p. 69). Even as speculation about ancestors was going out of fashion, many biologists agreed on the merits of “[t]his exquisite form” (H. F. Osborn in Willey 1894, p. ix).

Encyclopaedias acknowledged that some scientists put the “stem-father of all vertebrates” in a degenerate line (*Meyers Großes Konversations*-*Lexikon* 1905, pp. 454–455). Yet as research on amphioxus produced a huge volume of articles, books and models (e.g., Hatschek 1881; Willey 1894; Hopwood 2002, pp. 34–35, 101, 146), it thrived as (a relative of) an ancestor and for other reasons too. Accessible on various coasts, if hard to culture, it featured prominently in surveys (Balfour 1881, pp. 1–7; Korschelt and Heider 1892, II, pp. 1429–1467). Because “all the fundamental structures of the body are laid down with schematic clearness”, it provided an “unrivalled” introduction to embryology and “a refuge to the perplexed embryologist” (Willey 1894, p. 104).

The “Amphioxus song”, produced in and for biological summer schools in the interwar US, rejoices in this classroom staple:

A fish-like thing appeared among the annelids one day;

It hadn’t any parapods or setae to display.

It hadn’t any eyes or jaws or ventral nervous cord,

But it had a lot of gill slits and it had a notochord.

Chorus:

It’s a long way from Amphioxus,

It’s a long way to us.

…

It was a long way from the German tradition and Reymond’s Haeckelism to these more technical, less historical verses. But laughter over Haeckel’s cult still echoes in biologists’ amused pride in an animal which, for all his efforts, never quite became a household deity or even a household word.^17^

This look at the public life of amphioxus in its early years of fame has sought, on the one hand, to do more justice to its dominance than accounts that have dwelled on controversy over the ascidian theory and then the diverse challenges to what long remained the dominant view. It has contributed, on the other, to appreciation of the aesthetic dimensions of Darwinism and of species choice. Running through some of the fiercest battles in the history of science are smiles at Haeckel’s excesses easing acceptance that he spoke a core of truth, ancestor-worship that self-consciously imitated a religious cult, and affection for Naples as the site of discoveries that revealed ancient history along the Mediterranean shore.

## Footnotes

1 Roger Highfield, “Body-shapes are ‘down to limbless, headless fish-like creature’”, *Telegraph*, 18 June 2008, http://www.telegraph.co.uk/science/science-news/3344789/Body-shapes-are-down-to-limbless-headless-fish-like-creature.html, last accessed 25 Sept. 2014.

2 “Songs about science VII: ‘It’s a long way from amphioxus’”, *Nature* News Blog, 19 June 2008, http://blogs.nature.com/news/2008/06/songs_about_science_vii_its_a.html; further: Maienschein (1994, pp. 465–466); Joe Felsenstein, “The amphioxus song, or, ‘It’s a long way from amphioxus’”, http://evolution.genetics.washington.edu/amphioxus/amphioxus.html, last accessed 9 Jan. 2014; and on evolution songs: Smocovitis (2009).

3 For example, Browne (2001), Neyer (2009); for species politics: Hopwood (2011). The present article builds on the discussion, with respect not to amphioxus but to Haeckel’s embryos, in Hopwood (2015).

4 For example, “Neue Literatur über Darwins Lehre von der Umbildung (Transmutation) der Arten”, *Ausland*, *41* (1868), 673–682, on 681–682; “Ernst Haeckels natürliche Schöpfungsgeschichte. 2. Die Stammbäume für Thiere und Pflanzen”, *Ausland*, *43* (1870), 710–715, on 712–713; “Darwin über die Abstammung des Menschen. III (Schluß)”, *Allgemeine Zeitung*, Nr. 125B (5. Mai 1871), 2214–2215; on Darwinism in the periodical press: Kelly (1981), Daum (1998, pp. 300–323), Hopwood (2015, pp. 99–105); for the context: Blackbourn (1997, pp. 270–283).

5 Zacharias (1875a); see also, e.g., “Häckel’s Entwickelungsgeschichte des Menschen. III”, *Weser-Zeitung*, Nr. 9995 (19. Nov. 1874, Morgenausgabe), 1–2; Zacharias (1875b).

6 Sterne (1875, p. 268); see also Bock (1875), and on the magazine, Belgum (1998). The journalist Ernst Krause (pseudonym Carus Sterne) tried to enlist Haeckel’s help with drawing—hard for a periodical that preferred natural-looking scenes to the didactic, textbook-like cuts in the *Illustrirte Zeitung*: “It would be most suitable for these purposes if, in a section, e.g., of the Naples aquarium we could unite into a group picture an ascidian rooted on the floor a[nd] in the water amphioxus a[nd] sea lamprey with the larvae of all three species of animals” (Krause to Haeckel, 19 Nov. 1875, Ernst-Haeckel-Haus, Jena). The article appeared without illustrations.

7 They thus added to criticism by the aged Karl Ernst von Baer, reported in Huber (1874).

8 Vogt (1875); “Ultradarwinismus und Dilettantismus. Aus der Laienperspective”, *Allgemeine Zeitung*, Nr. 105B (15. Apr. 1875), 1625–1626. Semper would object to Haeckel’s claim that he “had denied *any* relationship of amphioxus with the vertebrates” (Semper 1877, p. 35); Ernst Krause would regret that “poor amphioxus must share [Semper’s] hatred of you” (Krause to Haeckel, 30 Jan. 1877, Ernst-Haeckel-Haus, Jena).

9
*Deutsches Biographisches Archiv*, s.v. “Reymond, Moritz”; Hopwood (2015, pp. 138–143). Reymond (1880b) discussed, in part sympathetically, the antisemitism of Wilhelm Marr.

10 This was Haeckel’s spelling, and hence Reymond’s and Edward Chapman’s below.

11 “Der moderne Prometheus”, *Periodische Blätter zur wissenschaftlichen Besprechung der großen religiösen Fragen der Gegenwart*, *6* (Juli 1878), 92–100; M[üller] (1877).

12 W. Breitenbach quoted in a flyer for Reymond (1912).

13 Agorastes (1878). Wolfe (1966) does not list this first edition, but Chapman (1899, pp. 81–88) includes the verses.

14 Not included here are the marginal titles Chapman gave each verse, e.g., “Horrid advent of an anatomist”, or the one historical note. Though the subtitle referred to “missing links”, there is little of the anxiety sometimes associated with that term: Beer (1996, pp. 115–145). Chapman does not seem to have translated Straubinger’s song, which offered less lyrical potential.

15 I have touched the highest point—/And from that full meridian of my glory/I haste now to my setting.—Shakespeare. [Chapman’s note.]

16 Reymond (1880a, pp. 80–86). The reference was to praise for the *Schöpfungsgeschichte* in Darwin (1871, I, p. 4), too early really to have played this role.

17 More specialized poems communicated the research of the British zoologist Walter Garstang on the evolution of larval forms and criticized Haeckel’s doctrine of recapitulation: Garstang (1951).
